# Wrangle with hypertension: lowered salt intake may not compromise iodine status among tribes of Central India

**DOI:** 10.1017/S1368980022000131

**Published:** 2022-04

**Authors:** Tapas Chakma, Suyesh Shrivastava, Arvind Kavishwar

**Affiliations:** 1 Division of Non-Communicable Diseases, ICMR-National Institute of Research in Tribal Health, PO-Garha, Nagpur Road, Jabalpur 482003, MP, India; 2 Principal Technical Officer, Division of Non-Communicable Diseases, ICMR-National Institute of Research in Tribal Health, Jabalpur, MP, India

**Keywords:** Hypertension, Urinary Iodine, Tribals

## Abstract

**Objectives:**

The most important risk factor of cardiovascular disease is hypertension and high salt intake contributes to high blood pressure. However, to prevent iodine deficiency disorders, the iodisation of salt is a proven strategy. So, on one hand, we suggest people reduced salt consumption but on the other hand, we also fear an increase in the prevalence of iodine deficiency disorders. In the present study, we investigated the possibility of salt intake at WHO recommended levels resulting in higher or lower iodine status in India by assessing the urinary iodine status and its relation with blood pressure.

**Design:**

It was a cross-sectional study.

**Setting:**

It was a community-based study.

**Participants:**

We collected 24-hour urine samples for estimation of iodine concentrations in urine from 411 adult hypertensives in the Mandla district of central India. Urinary iodine was estimated using Thermo ORION make ion-selective electrodes.

**Results:**

The median urinary iodine excretion was 162·6 mcg/l. Interestingly 371 (90·26 %) subjects were observed with > 200 mcg/l urinary iodine concentration level indicating iodine sufficiency. Individuals with high urine Na significantly had high blood pressure as compared with individuals with low urinary Na excretion (*P* < 0·01). There is a higher probability of high urine iodine levels among individuals with higher urine Na levels.

**Conclusion:**

The study revealed that 90 % of the population were excreting excessive iodine in urine, which is more than adequate iodine uptake. This excess uptake enables a scope for reduction in salt intake to control hypertension.

According to the global health report, chronic diseases have emerged as a major challenge to public health^(1,2)^. Globally noncommunicable diseases alone account for 63 % (36 million) of all deaths annually. The majority (80 %) of these deaths occur in poor countries^(3)^. In India, a rapid health transition occurs due to a rapid increase in the burden of non-communicable diseases in comparison with communicable diseases per se. An estimated six million deaths occurred in India in 2016 due to one or other non-communicable diseases^(4)^.

The most significant modifiable risk factor of CVD is hypertension (HTN)^(5)^. It is reported that high Na intake, that is more than 2 g/d which is equivalent to about 5 g salt/d, and low potassium intake, that is less than 3·5 g/d contributes to high blood pressure and increases the risk of various heart diseases significantly. Salt in our daily diet is the main source of Na^(6)^. According to WHO^(7)^, individuals mostly consume about 9–12 g salt/d, which is twice the recommended level of 5 g/d.

In India, the average daily salt intake is 8–12 grams per person per day^(8–11)^.

To reduce CVD-related morbidity and mortality, several strategies were implemented. Among all, reducing dietary Na has been included in many guidelines such as in WHO^(7)^, National Program of India for the treatment of HTN. WHO’s SHAKE technical package is a prescribed model for the reduction of salt intake in communities. The package is successfully adopted by many countries. Adopted countries have agreed to reduce the population’s intake of salt by 30 % by 2025^(7)^. But, reduction of salt intake to control HTN has been opposed by experts involved in iodine deficiency disorder^(12)^. Further, a few studies have demonstrated a positive correlation between low salt intake and higher risk of HTN or cardiovascular events^(13,14)^. Moreover, prolonged practice of low salt increases blood pressure through activation of the renin–angiotensin and sympathetic nervous systems^(13)^.

Currently, low-Na diet is an integral part of HTN management. However, concerns raised by a few experts of iodine deficiency disorders regarding the possible reduction of iodine intake during salt restriction remain. Iodine is an essential micronutrient for the body and its major source for the Indian population is iodised salt. Presently data showing the correlation between iodine level, blood pressure and other factors is lacking, particularly among the tribal population of central India. The present studies were carried out to address a few of these issues in the Mandla district of Madhya Pradesh (Fig. 1) .

## Objective

The main objective was to assess the urinary iodine status and its relation with blood pressure.

## Methodology

This present study was embedded in a larger study titled ‘Assessing Prevalence of Hypertension in Relation to Urinary Excretion of Sodium Among Tribal Adult Population in the District of Mandla, Madhya Pradesh’ in sampled villages and urban wards. Detailed methodology is discussed in the original article^(15)^.

To establish the iodine status, an estimation of 24-h urinary iodine concentration (UIC) was done among 411 subjects from Mandla district. There are six tehsils and nine developmental blocks in Mandla. As per census^(16)^, there are a total number of 1 054 905 dwellers residents in the district. A total of 411 adult human samples were included for UIC analysis. This method uses an estimation of 24-h Iodine excretion or evaluating iodine levels in the body. All consenting adults more than 20 years and residing in the study area continuously for more than a decade were included. Visitors and guests were excluded from the study along with the pregnant women.

### Blood pressure measurements

Blood pressures were measured according to the method described by Chakma *et al.* ^(15)^.

### Urinary iodine estimation

Twenty-four-hour urine sample was collected as per Chakma *et al.* ^(15)^ for estimation of urinary iodine. Samples were collected in screw-capped plastic containers, transported to the laboratory and stored at 4–8°C till analysis. The 24-h UI collection is considered the ‘reference standard’ for the measurement of the iodine intake in an individual^(16)^.

Urine iodine was estimated using an Ion meter (Thermo Orion) at ICMR National Institute for Research in Tribal Health (NIRTH), Jabalpur and was expressed as mcg of iodine/l of urine (mcg/l). Urine iodine level < 100 mcg/l is considered as insufficient iodine, 100–200 was adequate, > 200–300 was more than adequate and >300 mcg/l is considered as excessive iodine intake (Table 1).

**Fig. 1:**
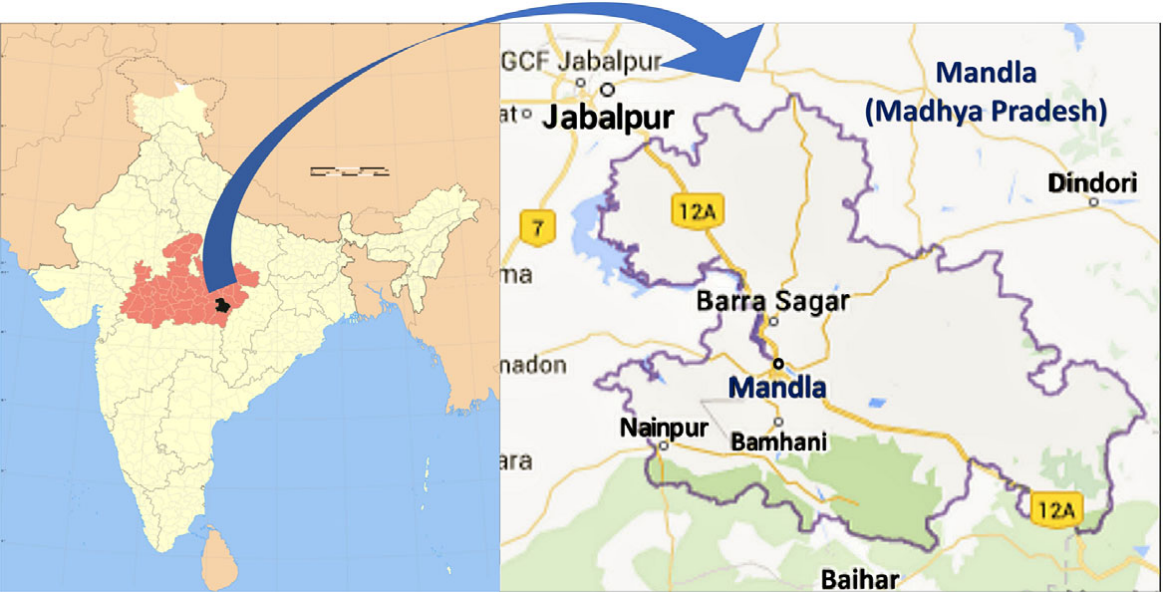
Map of Mandla district (study area)

**Table 1:** Epidemiologic criteria for assessing iodine nutrition based on median urinary iodine concentrations in different target groups*

Urine iodine mcg/l	Iodine intake	Iodine nutritional status*
< 100	Insufficient	Deficiency
100–199	Adequate	Optimal
200–299	More than adequate	Risk of iodine-induced hyperthyroidism
300	Excessive	Risk of autoimmune thyroid disease

* WHO/NHD/01·1 s edition, 2001.

Urine Na was estimated by a colorimetric method and using the commercially available kit (Accucare urine reagent strips (Catalog No. = URLX 100), Lab-Care Diagnostics Pvt. Ltd, Mumbai, India). This method is based on the reaction of Na with a selective chromogen producing a chromophore whose absorbance varies directly as the concentration of Na in the test specimen. The range of < 0·2–260 mmol per liter (mmol/l) was considered normal.

### Statistical analysis

χ^2^ test was used to study the magnitude of the association between UIC and age groups, gender, blood pressure and BMI categories. Association of urinary Na and blood pressure was also seen similarly. OR were computed. The critical level of significance was two-tailed *P* < 0·05.

## Results

A total of 411 sampled individuals (194 male and 217 female), participated and provided 24-h urine samples. A total of 98 % of individuals were using iodised salt. The median UIC was 162·6 mcg/l. Interestingly, 371 (90·26 %) subjects were observed with UIC > 200 µg level. The average age of individuals with UIC > 200 mean age was 39·89 ± 11·4 years and those with UIC < 200 had a mean age of 42·30 ± 12·02 years. Raised UIC was significantly associated with higher diastolic blood pressure (*P* < 0·05), OR = 1·92 (95 % CI 0·997, 3·702) suggesting that raised blood pressure may be due to high salt consumption resulting in raised peripheral resistance and consequently high diastolic blood pressure.

Table 3 shows a significant and positive association of urinary Na with HTN. About 71 % of individuals with high urine Na (≥ 260 mmol/24-h) had high blood pressure as compared with 50 % among individuals with low urinary Na excretion (< 260 mmol/24-h) individuals. The difference was statistically significant (χ2 = 9·96, df = 1, *P* < 0·01).

The BMI also showed slightly higher with UIC > 200, although statistically, this was insignificant. However, 95 % CI showed a higher probability (15·3 %) of raised BMI and higher UIC (Table 2).

**Table 2:** Distribution of 24 h urinary iodine excretion in relation to various factors in Mandla Madhya Pradesh

Factors	< 200 mcg/l (*n* 40)	%	> 200 mcg/l (*n* 371)	%	Significance, OR	95 % CI	*P* value
Age < 40	17	42·5	182	49·1	χ^2^ = 0·6215, OR = 0·7676	0·3971, 1·484	*P* = 0·4305
> 40	23	57·5	189	50·9			
Mean	42·30		39·89		t = 1·258	-1·1354, 6·169	*P* = 0·209
sd	12·02		11·44				
Male	23	57·5	171	46·1	χ^2^ = 1·886, OR = 1·582	0·8185, 3·059	*P* = 0·1699
Female	17	42·5	200	53·9			
SBP < 120	17	42·5	108	29·1	χ^2^ = 3·059, OR = 1·8	0·925, 3·502	*P* = 0·08032
SBP > 120	23	57·5	263	70·9			
DBP < 80	20	50·0	127	34·2	χ^2^ = 3·908, OR = 1·921	0·9972, 3·702	*P* = 0·0480*
DBP > 80	20	50·0	244	65·8			
BMI < 25	38	95·0	312	84·1	χ^2^ = 3·396, OR = 3·593	0·8438, 15·3	*P* = 0·06536
BMI > 25	2	5·0	59	15·9			

SBP: systolic blood pressure, DBP: diastolic blood pressure.

Table 4 describes the relationship of between urinary Na and UIC. A total of 97 % of samples with high urine Na also have high UIC as compared with 89·8 % samples with normal urine Na levels. Though the difference is not statistically significant (*χ^2^ = 1·835, *P* > 0·05, OR = 3·674, lower limit (LL) = 0·6697, upper limit (UL) = 77·63), it indicates that there is a higher probability of higher iodine levels among the higher urine Na level as the upper limit of 95 % CI is 77·63.

**Table 3:** Distribution of 24 h urinary sodium in relation to hypertension

Blood pressure	Urine Na < 260 mmol	%	Urine Na > 260 mmol	%
Normal	174	49·6 %	18	30·0 %
Hypertensive (systolic blood pressure > 140 mmHg)	177	50·4 %	42	70·0 %*
Total	351	100 %	60	100 %

* χ^2^ = 7·885, *P* < 0·01, OR = 1·746, 95 % CI (LL = 0·6112, UL = 2·539).

**Table 4:** Distribution of 24 h urinary Na in relation to urinary iodine concentration (UIC)

UIC (mcg/l)	Urine Na < 260 mmol	%	Urine Na > 260 mmol	%
< 200	39	10·3 %	1	3·0 %
> 200	339	89·7 %	32	97·0 %*
Total	378	100 %	33	100 %

* χ^2^ = 1·835, *P* > 0·05, OR = 3·674, LL = 0·6697 UL = 77·63.

## Discussion

We observed that higher urinary Na and UIC were proportionately related to high blood pressure. We also observed in another study that salt uptake was directly associated with high blood pressure^(14)^. Our findings were supported by Surya Bali *et al.* ^(18)^. They reported similar findings among school children of adjacent district Jabalpur where the median UIC level was 218 mcg/l. Our findings were also supported by community-based surveys reported by Lohiya *et al.* ^(19)^. They reported that 73 % rural population of Haryana was using iodised salt (≥ 15 ppm). Iodine was deficient in only 17 % respondents (UIC < 100 mcg/l). Similar results were also shown by Charlton *et al.* ^(20)^ who reported median UIC was 120 mcg/l.

Earlier guidelines have indicated that excessive (> 300 mcg/l per day) Iodine consumption may be harmful particularly in areas where iodine deficiency has previously been reported. Thence they may be prone to adverse health consequences, like iodine-induced hyperthyroidism, etc^(21)^.

Katagiri *et al.* ^(22)^ reported that universal salt iodisation although has improved goitre rates, but it simultaneously may lead to hypothyroidism due to excess iodine from excess salt.

### Limitations

The current estimates of iodine nutrition from Mandla district of Madhya Pradesh are based on the UIC carries several shortfalls like a small set of data, underestimation or overestimation on the extent of iodine excess or deficiency.

### Strength of the study

The urine samples are a subset of a large population of 1258 adults screened for HTN and other factors drawn from 33 sample villages/urban wards through probability proportion to population size sampling. Moreover, these were 24-h urine samples collected from the community.

## Conclusions

The study revealed that 90 % of the population were excreting excessive Iodine in urine, it indicates more than adequate iodine uptake. This excess uptake enables scope for reduction in salt intake to control HTN. However, we also recommend adopting the SHAKE package for the country.
